# New Perspectives on Oxidized Genome Damage and Repair Inhibition by Pro-Oxidant Metals in Neurological Diseases

**DOI:** 10.3390/biom4030678

**Published:** 2014-07-17

**Authors:** Joy Mitra, Erika N. Guerrero, Pavana M. Hegde, Haibo Wang, Istvan Boldogh, Kosagi Sharaf Rao, Sankar Mitra, Muralidhar L. Hegde

**Affiliations:** 1 Department of Radiation Oncology, Houston Methodist Research Institute, Affiliate of Weill Medical College of Cornell University, 6550 Fannin St, Smith 8-030, Houston, TX 77030, USA; E-Mails: jmitra@houstonmethodist.org (J.M.); enguerrero@houstonmethodist.org (E.N.G.); pdixit@HoustonMethodist.org (P.M.H.); hwang@houstonmethodist.org (H.W.); smitra2@houstonmethodist.org (S.M.); 2 Houston Methodist Neurological Institute, Houston, TX 77030, USA; 3 Centre for Neuroscience, Institute for Scientific Research and Technology Services (INDICASAT-AIP), City of Knowledge, P.O. Box 0843-01103, Panama; E-Mail: kjr5n2009@gmail.com; 4 Department of Biotechnology, Acharya Nagarjuna University, Nagarjuna Nagar, Guntur 522510, India; 5 Department of Microbiology and Immunology, University of Texas Medical Branch, Galveston, TX 77555, USA; E-Mail: sboldogh@utmb.edu

**Keywords:** redox transition metals, heavy metals, DNA base excision repair, metal toxicity, metal homeostasis, neurodegeneration, Alzheimer’s disease, Parkinson’s disease

## Abstract

The primary cause(s) of neuronal death in most cases of neurodegenerative diseases, including Alzheimer’s and Parkinson’s disease, are still unknown. However, the association of certain etiological factors, e.g., oxidative stress, protein misfolding/aggregation, redox metal accumulation and various types of damage to the genome, to pathological changes in the affected brain region(s) have been consistently observed. While redox metal toxicity received major attention in the last decade, its potential as a therapeutic target is still at a cross-roads, mostly because of the lack of mechanistic understanding of metal dyshomeostasis in affected neurons. Furthermore, previous studies have established the role of metals in causing genome damage, both directly and via the generation of reactive oxygen species (ROS), but little was known about their impact on genome repair. Our recent studies demonstrated that excess levels of iron and copper observed in neurodegenerative disease-affected brain neurons could not only induce genome damage in neurons, but also affect their repair by oxidatively inhibiting NEIL DNA glycosylases, which initiate the repair of oxidized DNA bases. The inhibitory effect was reversed by a combination of metal chelators and reducing agents, which underscore the need for elucidating the molecular basis for the neuronal toxicity of metals in order to develop effective therapeutic approaches. In this review, we have focused on the oxidative genome damage repair pathway as a potential target for reducing pro-oxidant metal toxicity in neurological diseases.

## 1. Introduction

Metal ions, many of which are essential in trace amounts, were also the first toxins known to humans. Even the essential metals are toxic or could be fatal when present in excess. A common mechanism of toxicity caused by redox-active metals is via the generation of reactive oxygen species (ROS), which affect both DNA and proteins.

While metals have been implicated in multiple pathologies, including cancer, neurotoxicity is believed to be their most common manifestation. Compelling evidence has etiologically linked abnormal brain metal accumulations with aging, as well as various neurological disorders, including Alzheimer’s disease (AD), Parkinson’s disease (PD), amyotrophic lateral sclerosis (ALS), Wilson’s disease (WD) and stroke [1,2,3,4]. Furthermore, only a fraction of these metal-accumulation diseases can be sourced to dietary or occupational exposure [1,5,6,7], although the source, as well as precise mechanism(s) of the majority of metal accumulation conditions remain obscure. It is important to mention that unlike other toxic compounds, the increased metal ion level could impact the homeostasis of all metals in the cell or organ due to its charged state and the natural cellular response towards maintaining a tight charge balance. In spite of an extensive literature on individual metal toxicity, the impact of metal dyshomeostasis is not completely understood.

Because of extensive genome damage observed in metal-associated diseases, a casual linkage was suggested between excessive cellular metal levels and genotoxicity. Subsequent studies established the role of heavy metals, such as lead (Pb), chromium (Cr), nickel (Ni), *etc.*, in causing direct damage to DNA via cross-linking and interference with transcription and replication processes, enhancing mutagenesis. Other metals, including cadmium (Cd) and copper (Cu), were shown to hinder cell growth and promote apoptosis [8], although, the underlying mechanism is not clear.

Interestingly, recent studies have implicated a reduced genome damage repair capacity in neurons of individuals afflicted with metal-accumulating neurodegenerative diseases [9,10,11], implicating the accumulation of pro-oxidant metals in enhanced levels of unrepaired oxidative DNA damage. This prompted us and other investigators to examine the effect of metals on DNA repair pathways and to understand the interplay among these diverse etiologies in neuronal toxicity. Redox-active Cu and iron (Fe) not only induce genome damage, but also specifically inhibit key enzymes in the oxidative genome damage machinery by both altering their structure and reversibly oxidizing cysteine residues in these proteins. We propose that inhibition of DNA repair plays a critical role in affecting the survival of postmitotic neurons, with inherently limited capacity for genome maintenance, because of the absence of replication-coupled repair processes. Furthermore, the reversible nature of metals’ inhibitory effect on DNA repair could be exploited to restore repair using specific metal chelators with reducing activity. These studies, which were subsequently corroborated by others, provided a new paradigm in our understanding of metal genotoxicity in neurons. In this review, we discuss the current knowledge on DNA repair pathways in neurons, the role of both essential and toxic metals in blocking these processes and how the mechanistic understanding of the phenomenon could be exploited for efficient therapeutic interventions.

## 2. Oxidative Insults and Repair in Human Genome

Oxidative genome damage is an inevitable consequence of metabolism in aerobic organisms. ROS and reactive nitrogen species (RNS) are continuously produced during respiration [12] and also during the activation of cellular defense pathways [13]. The levels of these pro-oxidants, including free radicals, can be enhanced several fold upon exposure to external factors, like heavy metals, smoking, ionizing radiation and other genotoxic stress [14,15,16]. A balance between DNA damage and repair capacity exists in a healthy cell to efficiently repair an estimated 10^4^ oxidized base lesions and single-strand breaks (SSBs) that are generated by ROS daily in a mammalian cell’s genome [17,18]. In addition to ROS and RNS, accumulated transition metals (e.g., Fe, Cu, Cr, Cd and cobalt (Co)), most of which are redox-active, induce a plethora of toxic free radicals through their participation in various cellular oxidation-reduction reactions. In the presence of hydrogen peroxide, these Fe and Cu ions generate toxic hydroxyl radicals via a Fenton or Fenton-like reactions [19,20], as illustrated below.

Fe(III) + O_2_^●−^ ↔ Fe(II) + O_2_

Fe(II) + H_2_O_2_ → Fe(III) + OH^●^ + OH^−^ (Fenton reaction)

Cu(I) + H_2_O_2_ → Cu(II) + OH^●^ + OH^−^

In addition, hydroxyl free radicals are also generated as a product of the Haber–Weiss reaction [21].

O_2_^●−^ + H_2_O_2_ ↔ O_2_ + OH^●^ +OH−

OH**^●^** + H_2_O_2_ → H_2_O + H^+^ + O_2_^●−^

### 2.1. Types of Oxidative Damage in DNA

**Figure 1:**
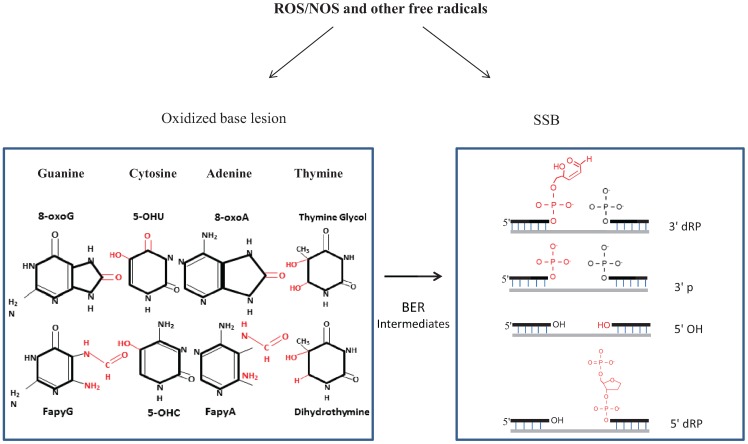
ROS and RNS are constantly produced during aerobic metabolism. ROS generate several dozen oxidized base lesions and single-strand breaks (SSBs) in the genome. SSBs are also generated as intermediates during the processing of oxidized bases via base excision repair (BER). ROS-induced SSBs contain diverse termini, like 3'-phosphoglycolate, 3'-phosphate, 5'-OH and 5'-deoxyribose phosphate.

Several dozens of oxidized base modifications, apurinic/apyrimidinic (AP) lesions, oxidized sugar fragments and SSBs with various blocked termini (Table 1) are induced by ROS in the human genome [22,23]. These, when unrepaired, have been implicated in various pathophysiological conditions. Common base lesions 5-hydroxyuracil (5-OHU), thymine glycol, 8-oxo-7,8-dihydroguanine (8-oxoG), 2,6-diamino-4-hydroxy-5-formamidopyrimidine (Fapy-G) and 4,6-diamino-5-formamidopyrimidine (Fapy-A) have been identified in affected regions of the AD brain (Figure 1) [24,25]. Sugar oxidation normally causes SSB formation in DNA [26,27], which is also formed as an intermediate during the repair of oxidized base lesions. The common ROS-induced SSBs include 3'-phosphoglycolate, 3'-phosphate and 5'-deoxyribosephosphate blocked termini, whose processing is a critical step in the repair pathway [28,29].

**Table 1:** Human diseases associated with metal toxicity. While excessive metal ions have been linked to genomic instability in cancer, the major manifestation of metal toxicity is aging and age-related neurological diseases, implying the susceptibility of the CNS.

Disease	Linked Metal Toxicity	References
Alzheimer’s Disease	Fe, Cu, Zn, Al	[30,31,32,33,34]
Parkinson’s Disease	Fe, Zn, Al, Cu, Mn	[1,10,34,35,36,37,38,39,40]
Huntington’s Disease	Cu, Fe	[41,42,43,44,45,46,47]
Wilson’s Disease	Cu, Fe	[48,49,50,51]
Amyotrophic Lateral Sclerosis	Fe, Cu	[52,53]
Friedreich’s Ataxia	Fe, Cu, Zn	[54,55,56]
Xeroderma Pigmentosum	Co, Cd, Ni	[57,58,59]
Cancer	Fe, Pb, Cd, Ni, Hg, Co	[20,60,61,62,63,64,65]

### 2.2. Repair of Oxidative Genome Damage: Basic Mechanism(s) and Complexity

Most oxidized damage in the genome, including base lesions, AP sites and SSBs, are repaired via the base excision/single-strand break repair (BER/SSBR) pathway, which is highly versatile and evolutionarily conserved from bacteria to humans. In its simplistic form, BER involves four key reactions, namely: (i) recognition and excision of the base lesion by a DNA glycosylase and cleavage of the resulting AP site by the glycosylase itself (for oxidized base-specific bi-functional enzymes) in a sequential reaction or by AP endonuclease 1 (APE1) (for mono-functional glycosylases) in the following step; (ii) processing of the 5' and/or 3' blocked termini by specific SSB end cleaning enzymes to generate polymerase-compatible 5'-P/3'-OH ends; (iii) nucleotide gap filling by a DNA polymerase; and finally, (iv) DNA ligases (Lig III or I) seal the resulting nick to complete the repair process.

Oxidized base-specific DNA glycosylases, discovered so far in humans, belong to two families, namely Nth and Nei. 8-oxoguanine DNA glycosylase-1 (OGG1) and NTH1 in the Nth family prefer oxidized purines and pyrimidines, respectively, from duplex DNA [66]. Recently discovered NEIL1 and NEIL2, belonging to the Nei group [67,68,69], mostly prefer oxidized pyrimidine, both in duplex and single-stranded DNA, with a weaker activity with some purine lesions [70,71]. Recently characterized NEIL3, the fifth member of the Nei family [72], was shown to remove a broad spectrum of base lesions on single-stranded DNA with a preference for spiroiminodihydantoin (Sp) and guanidinohydantoin (Gh), which are the subsequent oxidation products of 8-oxoG [73,74]. Two types of 3'-non-ligatable termini are generated during the processing of oxidized bases by these glycosylases. OGG1 and NTH1 execute a β lyase reaction at the AP site yielding 3'-phospho-α,β-unsaturated aldehyde (3'-dRP) and 5'-P; on the other hand, NEILs catalyze βδ elimination, forming 3'-P and 5'-P groups [75]. APE1 and polynucleotide kinase 3'-phosphatase (PNKP) work downstream of glycosylases in the BER pathway, removing 3'-dRP and 3'-P, respectively. However, APE1 primarily processes AP sites generated by mono-functional glycosylases to produce strand breaks with 3'-OH and 5'-dRP ends. In mammalian cells, Polβ acts as a bi-functional enzyme, possessing both polymerase and 5'-dRP lyase activity, removing the dRP residue to generate 5'-OH terminus [76], in addition to incorporating the missing nucleotide at the 3'-OH. While Polβ predominantly participates in single nucleotide incorporation or short patch (SP- or SN-) BER, replicative DNA polymerase δ (Polδ)-mediated BER was identified to involve a repair patch size of 2–8 nucleotides in a long patch (LP) BER pathway. Flap endonuclease 1 (FEN-1) is required for such repair to cleave off the displaced flap structure [77,78]. Furthermore, although few studies have suggested the ability of Polβ to participate in LP-BER *in vitro*, in coordination with FEN-1, whether it occurs *in vivo* is unclear [79]. In the final repair step, LigI or LigIIIα complete the nick sealing process of the phosphodiester backbone. LigI primarily acts during DNA replication and during LP-BER [23,80]. Recently, LigIII was shown to be redundant for nuclear DNA repair, because LigI serves as proficient back up [81]. The LigI expression level is highest in proliferating cells and reduced levels are present in non-cycling cells, like neurons, indicating that LigIII cannot substitute for all of the LigI-related activities [82]. A unique feature of the mammalian BER/SSBR is that the interactions of the BER proteins are highly coordinated and play a crucial role in efficient repair [78,83].

### 2.3. SSBR, a Variant of BER with Diverse End-Processing Reactions

ROS-induced SSBs could involve several types of non-ligatable termini, in addition to the ones observed in BER, and thus, SSBR employs additional end-processing enzymes [23]. 3'-phosphoglycolate and 3'-phosphoglycolaldehyde in mammalian genomes are generally removed by APE1. Tyrosyl phosphodiesterase I (TDP1) cleaves tyrosine-linked 3'-P generated by abortive topoisomerase I (Top I) reaction to 3'-P at the strand break in the TDP1/DNA complex [84,85]. Here, PNKP plays a dual role by both processing 3'-P and adding phosphate to the 5'-OH end [86]. Aprataxin releases 5'-AMP to restore the 5'-P terminus [87,88]. Some 3'-non-ligatable termini could also be excised by the XRCC1/XPF nuclease complex, which has an essential role in the nucleotide excision repair (NER) pathway by incising the damage-containing DNA strand 3' to the lesion site. After the conventional 3'-OH/5'-P are generated, SSBR and BER follow the same gap filling and ligation reactions. Thus, although end processing is more diverse in SSBR than BER, they share the last two reaction steps and enzymes.

### 2.4. Complexity of BER/SSBR Pathway

Unlike in bacteria, BER/SSBR in human cells is highly complex, involving additional cellular proteins that regulate the cell cycle dependence of repair, genome region-specific repair sub-pathways and in several back-up pathways [77,89]. These include accessary proteins XRCC1, PARP-1 and -2, and non-canonical proteins hnRNP-U, YB1, *etc.* [23,75,89,90,91,92,93,94,95,96,97]. Furthermore, post-translational modifications in one or more components, including acetylation [98,99,100,101], phosphorylation [102,103,104], ubiquitylation [105,106,107], SUMOylation [108,109,110], methylation [111,112] and PARylation [113,114,115], regulate the repair process and rate in various cellular states. While a detailed account of specific modifications in BER/SSBR machinery is beyond the scope of this review, recent studies linking defects in acetylases/deacetylases with dramatic BER/SSBR deficiency and mutagenicity highlight the importance of such modifications in repair [101,116,117].

The presence of non-conserved, mostly disordered terminal peptide segments in early BER/SSBR proteins, as comprehensively reviewed in our previous review [23,118], could be critical for key functions, including damage sensing, protein-protein interactions, repair regulation via posttranslational modifications and nuclear localization signal (NLS) [118]. We believe that mammalian early BER enzymes, like NEIL1 or APE1, could be considered as “hub” proteins with multiple interacting partners (>10), most of which utilize terminal disordered segment as common interaction interface [23,118,119].

### 2.5. Mitochondrial (Mt) BER/SSBR

MtDNA is highly susceptible to oxidative stress because of its close proximity to the site of ROS generation and lack of protective histones [120,121]. The proteins involved in mtBER are encoded by the nuclear genome, many of which are identical to their nuclear counterparts, but few distinct mitochondrial specific isoforms exist. LigIII exists in distinct nuclear and mitochondrial isoforms, but only nuclear LigIIIα works in complex with the scaffold protein, XRCC1, *in vivo* [80,122]. Mitochondria have only one DNA polymerase (Polγ), which participates in both mtDNA replication and repair [123]. The high lipid content of mitochondrial membranes facilitates the accumulation of alkylating agents [124] and cationic metals, such as Cd, mercury (Hg), Pb, manganese (Mn), Fe and Cu [125,126,127]. Furthermore, as would be expected, a higher mitochondrial BER capacity is observed in brain than other tissue types [128,129].

## 3. Current Understanding of BER/SSBR in the Central Nervous System (CNS)

The CNS consists of the brain and spinal cord with multiple cell types, including neuron and glial cells. The major bottleneck for DNA repair in neuronal cells is that these cells are terminally differentiated and lack replication-associated repair [4]. Regulation of BER in neurons in various pathophysiological conditions is largely unknown [130]. During neuronal maturation, a decreased OGG1 level and increased NEIL1 and NEIL2 levels have been observed in rat brain [131]. It has been suggested that both NEIL1 and NEIL2 might have a potential role in maintaining the integrity of transcribed genes in terminally differentiated neurons. Studies demonstrating impaired memory retention in mice lacking NEIL1 further support the critical role of this enzyme in brain cells [132]. Besides protecting the genome from the accumulation of cytotoxic base lesions, the distribution and activity of NEIL3 in brain direct its specific role in stem cell differentiation [133,134]. NEIL3 is required for maintaining DNA integrity in neural progenitor cells, both during development and in adult brain [135]. NEIL3^−/−^ mice showed memory defects, coupled with the impaired proliferative capacity of hippocampal neural stem/progenitor cells, which is also correlated to reduced DNA repair capacity. Further, NEIL3-deficient neural progenitor cells were highly susceptible to oxidative stress [136]. Another glycosylase, specific for alkylated bases, MPG expresses strongly in cerebral cortex, cerebellum and hippocampus in brain [137]. Although, OGG1’s activity was found to be low in brain, NTH1 showed comparable expression and activity in brain, testes, kidney and liver. There are conflicting reports on brain OGG1 levels in ageing and neurological disorders: while a decreased OGG1 activity was observed in aged rat brain [138], its level was shown to increase in substantia nigra in PD [139]. Interestingly, a recent study suggested 8-oxoG repair via MUTYH-mediated BER (MUTYH excises A mispaired with 8-oxoG) rather than OGG1-mediated BER of the 8-oxoG-C pair. Consistently, mice lacking MUTYH were resistant to neurodegeneration, while mice lacking OGG1 or MTH1 (an enzyme that depletes 8-oxoGTP from the pool) exhibited severe striatal neurodegeneration [140]. These findings suggest that MUTYH-mediated 8-oxoG repair may promote neurodegeneration. Both PNKP and APE1 are highly expressed in brain. The neuroprotective role of APE1 in the survival of oxidative stress-induced post-mitotic neurons has been well established [141,142,143]. The interaction of PNKP with scaffold protein XRCC1 has been shown to be crucial for efficient BER processing during oxidative stress [144]. Polβ, the major DNA polymerase in non-cycling neurons, is ubiquitously expressed in all brain regions [145], and its level decreases in an age-dependent manner. Other DNA replication-associated repair proteins’, like FEN-1, Pol δ/ε, PCNA and LigI, levels have been observed to be low in the adult brain, as expected, while XRCC1 and LigIIIα levels are high with respect to other tissue types [146]. These studies indicate that SN-BER might be the major BER pathway with a reduced level of LP-BER in matured neurons [147,148].

## 4. Revisiting Metal Toxicity Diseases

### 4.1. Metal Accumulation in CNS Etiologically Linked to Neurological Diseases

Metals, such as Fe, Zn, Cu, Se, Mn, Mo, Co, Na and K, and other elements, like S, P, I, F, *etc*., are indispensable for life, because of their essential role in fundamental life functions. These are beneficial at a lower (optimum) concentration, but are toxic at a higher concentration. Most metals enter the brain through the “blood brain barrier” (BBB) using specific carrier proteins.

While the metal content is tightly regulated in normal brain, by being sequestered with storage proteins, e.g., ferritin, transferrin and ceruloplasmin, and released only in response to metabolic need, metal dyshomeostasis leading to the abnormal overload of free metal ions is a common feature in the pathogenesis of most, if not all, neurodegenerative diseases and aging. Specifically, accumulations of Fe, Cu, Al, Mn, *etc.* (either individually or together), is implicated in AD, PD, ALS, hereditary ferritinopathy and WD [48,149,150,151,152,153].

#### 4.1.1. Essential Transition Metals

Fe, Cu and Zn: The redox states of Fe(II/III) and Cu(I/II) that make them biologically important also make them toxic, due to the production of ROS. While most of the Fe in a healthy human body is present in hemoglobin, along with Cu and Zn, it serves as essential co-factors for a multitude of enzymes and signaling molecules and, thus, regulates various biological pathways, including mitochondrial energy production, DNA replication/transcription, synthesis of neurotransmitters, *etc.* In the normal brain, the major fraction of bio-available Fe and Cu (~70%) are typically sequestered in ferritin/transferrin and ceruloplasmin, respectively [154,155]. Studies have implicated Cu, Fe and Zn as key factors in the pathophysiology of AD and PD [156,157]. High levels of Cu (400 μM) and Zn (1 mM) were found in amyloid plaques and AD neuropil regions in comparison to healthy brain (70 μM Cu and 350 μM Zn) [32,158], which accumulate progressively during the transition from moderate to severe AD [156]. Furthermore, the injection of FeCl_3_ into the substantia nigra of adult rats resulted in a substantial selective decrease of striatal dopamine (95%), leading to PD-like symptoms [159]. Mn is another essential transition metal, ubiquitous in human cells in trace levels, primarily acting as a cofactor of mitochondria-specific SOD. Exposure of mice to a high Mn level causes a clinical disease characterized by extrapyramidal symptom resembling idiopathic PD (IPD) [160]. Further, the ability of Mn to enhance α-synuclein and amyloid-β misfolding/aggregation coupled with its abnormal distribution in brain suggests that Mn toxicity could contribute to AD, Huntington disease and ALS.

#### 4.1.2. Non-Essential Heavy Metals’ Impact on Human Health

Al, an abundant element in the environment, is used in industry, medicine, food, water processing and agriculture. The average uptake of Al(III) ranges from 2.5 to 13 mg/day, depending on the food and cooking practice of country. Although, consistently implicated in the pathogenesis of AD, for the better part of the last century, the definite etiology of Al toxicity in AD has remained unknown. Al has never been demonstrated to have any definite biological function, but many studies have brought to light its potential neurotoxicity in experimental animal models and in humans [161,162,163]. A detailed role of Al in neurodegenerative diseases was reviewed elsewhere [164].

Pb and Hg: Pb(II/IV) is a well-known neurotoxicant in children. The predominant route of exposure is through the ingestion of contaminated food, although Pd-based paint and contaminated water also contribute. Pb exposure has been linked with reduced IQ and behavior in children. It is considered to be a risk factor in accelerated cognitive decline, AD and PD [165]. Rodent studies suggest a reduction in the number of dopamine neurons and significant alterations in the nigrostriatal dopamine system after being exposed to Pb, which is consistent with PD pathology [166]. Pb also increases the expression of APP mRNA and elevated amyloid beta aggregation [167]. Primates exposed to Pb showed increased amyloidogenesis and senile plaque formation, both studies consistent with what is observed in AD brain samples [168].

Hg, another heavy metal, is highly neurotoxic, particularly when converted into methyl-Hg. This, commonly ingested via contaminated fish, passes through the BBB by binding to cysteine. Hg(II) exposure in humans causes PD-like pathology, including movement dysfunction, tremors and polyneuropathy. Mice exposed to methyl-Hg exhibit reduced neuritis of dopaminergic neurons and a reduction of striatal dopamine [169]. Finally, Hg and Pb could also generate ROS via lipid peroxidation, mitochondrial damage and superoxide production [170].

### 4.2. Complex Nature of Metal Toxicity in Neurodegenerative Diseases

#### 4.2.1. Environmental/Occupational Exposure *versus* Internal Redistribution

While occupational or environmental exposure to metals predisposes humans to neurological dysfunction, as expected, these constitute only a small fraction of human neurodegenerative diseases. The majority of patients showing a marked increase in the levels of various metals in brain have no known metal exposure or increased dietary intake. Interestingly, we demonstrated that metal levels in blood and brain are inversely correlated in AD and PD [171,172], indicating metal redistribution from blood to brain or CSF, but the mechanism is unclear. We proposed that such an increased metal uptake in the brain may be driven by a toxic misfolded (amyloid) protein response. Most metals promote amyloid protein aggregation. This makes the highly toxic misfolded amyloid proteins inert initially, but when the aggregate level increases beyond a cells’ threshold, it would aggravate the neurotoxicity.

#### 4.2.2. Transition Metals’ Charge-Dependent Changes with Disease Progression

Remarkably, studies also revealed that transition metals in their divalent states (e.g., Fe(II) or Cu(II)) increase in the early phase of AD, while trivalent forms (Fe(III) tend to increase in the later phase of the AD-affected brain [172]. Furthermore, an increase in trivalent metals forms in late-stage AD is accompanied with decrease in mono- or di-valent metals. Thus, the overall brain metal burden is never dramatically increased in AD; instead there is the abnormal redistribution of specific metals in different organs. In general, it appears that a common trait in many processes underlying neurodegeneration is a (direct or indirect) perturbation in the homeostasis of Cu, Zn, Fe, Al, *etc.* This could be important for formulating metal chelation therapy, which should consider supplementing the depleted metal, in addition to chelating the increased metal ions.

### 4.3. Metal Toxicity as Homeostatic Imbalance: A New Perspective

There is a limited database on trace metal homeostasis in brain. Although several reports are available on individual metal level changes/distribution, no studies have successfully correlated the inter-elemental relationships. Our limited previous studies provided new information on the metal-homeostasis pattern in the brain and serum samples of AD/PD patients [1,173]. Due to the tight regulation of charge balance in a cell and across the membrane, the effect of changes in the level of a particular metal is not restricted to that metal alone, but instead, it would impact many other metals in that particular organ and, eventually, lead to metal dyshomeostasis and cellular dysfunction. This information is novel, but needs substantial database development to compute a model reflecting the total trace elemental homeostasis in human brain. We feel that such a database will be of great significance, not only for the early diagnosis of neurodegenerative and neuro-psychiatric disorders, but also in understanding these disorders using elemental homeostasis as a model system. Although numerous hypotheses have been proposed for the involvement of metals in various neurological dysfunctions (reviewed in [7,174]), conclusive evidence still remains vague, presumably due to the complex and dynamic nature of metal levels and toxicity. Hence, there is a need for a methodology to map not only trace element levels, but also to establish metal-metal-dependent-interrelationships in human brain with relevance to progressive metal accumulation diseases.

## 5. Metals Induce Genome Damage, Both Directly and Circuitously

Metal toxicity-linked conditions, including most neurological disorders, are commonly associated with extensive genome damage, particularly oxidative base damage and strand breaks [175], which suggest a causal link between the two phenomenon. Redox cycling Fe and Cu generate ROS via the Fenton reaction producing hydroxyl and superoxide radicals that further damage DNA. These metals can also produce DNA damage by their directly binding and nicking activity [176]. Other heavy metals, like Pb, Cd, *etc.*, and Al also indirectly promote ROS/RNS generation by their high affinity binding to thiol groups containing enzymes and proteins [177,178], which are responsible for normal cellular defense mechanism against oxidative stress. Furthermore, while ROS and/or metals induce various oxidative modifications in DNA bases and SSBs, the accumulation of closely-spaced SSBs in non-cycling neurons could lead to secondary DSBs, which are the most toxic type of DNA genome damage [179].

## 6. Inhibition of DNA Repair by Redox Metals: A Double Whammy

### 6.1. Pro-Oxidant Metals Inhibit BER Pathways

While a significant decrease in genome repair capacity, including BER in neurons, has been observed in neurodegenerative diseases, as well as during ageing [180,181,182,183], the molecular events leading to such deficiency have not been characterized. A lack of a direct correlation between repair deficiencies with the expression of repair enzymes suggests the involvement of additional mechanisms. Repair defects involving heavy metal exposure were attributed to direct DNA binding, which not only induce DNA strand breaks, but also interfere with damage scanning and the recruitment of repair machinery [184,185]. However, repair defects in most cases of sporadic neurodegenerative diseases that involve the dyshomeostasis of essential transition metals, such as Cu and Fe, were not characterized until recently, when we demonstrated that Cu and Fe specifically inhibit the activities of NEIL1 and NEIL2 by forming stable complexes with these proteins [9]. We showed that Fe(II/III) and Cu(II) at nanomolar (nM) to low micromolar (µM) levels inhibit NEILs’ activity with various base lesion-containing DNA substrates *in vitro*. The inhibition was found to be very specific, as demonstrated by the lack of a similar inhibition of OGG1 with 8-oxoG substrates. This also suggested that in contrast to the previously suspected mechanism, the inhibition is due to metal binding to NEILs rather than to DNA. The binding involved both hydrophobic and electrostatic forces, as indicated by the large negative enthalpy. In cell inhibition of NEILs by these metals was also observed based on the reduced repair activity of NEILs in extracts from SH-SY5Y neuroblastoma cells treated with FeSO_4_ or CuCl_2_.

### 6.2. Mechanism of Repair Inhibition: Oxidation of Cysteines in NEIL DNA Glycosylases

Partial reversal of Cu-mediated NEIL1 inhibition by a Cu-chelator, but nearly complete reversal, by a combination of a Cu-chelator, plus a reducing agent or curcumin with dual metal chelation, as well as reducing properties, suggested that NEIL1 inhibition by Cu involves the reversible oxidation of cysteine residues [9]. Furthermore, the CD spectra of the NEIL1-Cu complex showed a small, but clear, change in the secondary structure of NEIL1, which could cause the inhibition of its activity. Interestingly, although both Cu(II) and Fe(II) bind to NEIL1 and NEIL2, the secondary structure of NEIL2, unlike NEIL1, is not affected significantly. Given the presence of a zinc finger motif in NEIL2, but not in NEIL1, Cu(II) or Fe(II) may competitively replace the intrinsic Zn ion in NEIL2 and compromise its folding.

Further, metal ions also disrupt protein-protein interactions during BER. DNA damage repair involves a highly coordinated signaling pathway that depends on appropriate protein-protein interactions during the signal transduction. We showed that Fe(II) affects NEIL1’s interaction with Polβ and FEN-1, which is critical for overall BER [9]. Thus, unlike previous studies that suggested that the inhibition of BER repair by metals may be due to their direct binding to DNA, these studies demonstrate a direct binding of redox metals with specific BER proteins, which affect their structure, as well as activities.

### 6.3. Summary of Metal Toxicity Impact on Other DNA Repair Enzymes/Pathways

In addition to NEILs, metal ions also inhibit other components of the DNA repair machinery (Table 2). It was observed that ~60% of LigIIIα activity was inhibited by FeCl_3_ (10–250 μM), which also affects Polβ [11]. The divalent ions, Cd^2+^, Ni^2+^ and Zn^2+^, inhibit the activity of recombinant human *N*-methylpurine-DNA glycosylase (MPG), a monofunctional DNA glycosylase, which removes a variety of alkylated bases [186,187]. Whiteside *et al.* showed that Cd and Cu inhibit phosphatase and kinase activities of both recombinant PNKP and its activity in human cell extracts [188]. Heavy metals could delay SSB rejoining in mammalian cells. Further, elevated Fe levels caused a reduction in FEN-1 and LigIII activities, due to the interference of repair protein binding to their DNA substrates [9].

**Figure 2:**
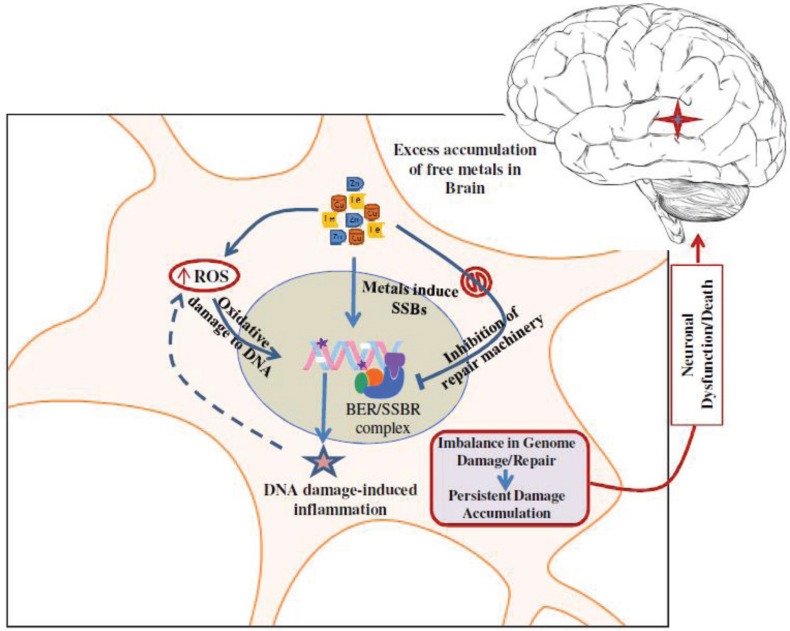
The genotoxicity of metals in neuronal cells: a “double-whammy”. Excess accumulation of metals in neurons not only causes increased oxidative damage to the genome, both directly and via ROS, but also inhibits the repair of such damage by binding/oxidizing certain repair proteins. This creates an imbalance between genome damage and repair, and the resulting persistent accumulation of damage contributes to neuronal dysfunction and cell death.

Thus, metal mediated genotoxicity could involve the induction of oxidative genome damage via: (i) free radical generation; and (ii) direct DNA binding; and inhibition of DNA repair via (i) inhibition of key BER/SSBR enzymes; and (ii) inhibiting interactions among repair proteins. Taken together, these studies showed that excessive Fe/Cu and other pro-oxidant metals in neurodegenerative brains act as a “double whammy” by inducing ROS that damage the genome and also by inhibiting damage repair at the same time (Figure 2).

**Table 2:** Key genome repair enzymes affected by metals. Transition and heavy metals have been shown to inhibit the repair activities of key proteins involved in the BER, single-strand break repair (SSBR) and DSBR pathways.

Repair Protein Affected by Metal(s)	Repair Pathway	Inhibiting Metal	References
NEIL1	BER	Fe, Cu	[9,175]
NEIL2	BER	Fe, Cu	[9,175]
APE1	BER/SSBR	Fe, Cd, Pb	[189]
PNKP	BER/SSBR/DSBR	Cd, Cu	[188]
FEN-1	BER/SSBR/DSBR	Fe	[11]
LigIII	BER/SSBR/DSBR	Fe	[11]
MPG	BER	Cd, Ni, Zn	[186,187]

## 7. Conclusions and Future Perspectives

### Molecular Understanding of the Complex, Multi-Targeted Nature of Metal Toxicity Is Important for Intervention Strategies

Despite an enormous increase in our understanding of the neuropathological events in aging and in ~200 types of neurological conditions, there is no cure available; current treatments slow progressive dementia only temporarily. This underscores the necessity of an overarching approach to explore newer strategies to unravel the mechanism of initiation/progression of neurodegeneration and to find more effective ways to prevent its onset, delay the progression and then effectively treat the disease to improve the quality of life for patients and their caregivers. Furthermore, despite multiple studies providing compelling evidence linking the misfolding/oligomerization of Aβ/tau, the build-up of Fe/Cu and other metals, the accumulation of genomic damage in the brain, how the interplay among these factors triggers neuronal apoptosis is still unclear. The role of metals as the key mediators of AD pathology has been reinforced by the fact that metal-chelation therapy has turned out to be promising in animal models of AD, although challenges regarding chelator choice and/or dosage together with the side effects of their sequestration of essential metals persist [9,190]. Our studies demonstrating that Fe/Cu acts as a “double whammy” by inducing genome damage and also inhibiting its repair provided a molecular basis, whereby Cu reduces cysteine residues in NEILs, which could be reversed by a Cu chelator in combination with a reducing agent or curcumin, which has both chelating and reducing properties. In addition, other compounds/drugs, particularly of natural/plant origin with antioxidant/free radical scavenging, as well as metal chelation activities, should be looked into for their neuroprotective functions [191]. For example, melatonin, which possesses both antioxidant and metal chelation properties, has been demonstrated to reduce metal-induced toxicity in cells [192]. However, the protective role of melatonin in preventing metal-induced DNA repair inhibition still needs to be investigated. In view of melatonin’s ability to stimulate antioxidant enzymes, in addition to free radical scavenging and metal chelation, it will be interesting to examine a combination of melatonin and curcumin for preventing multimodal metal toxicity conditions. These results suggested that it is important to comprehensively understand the metal levels in a given case; characterizing the complete metal homeostasis and specific toxicity of the metals involved, including their role in genome damage/repair systems is required in order to develop an effective therapy [191]. This underscores the need to re-examine the role of metal toxicity in neurological diseases as a part of a therapeutic strategy in light of the new information of the human genome and its functions.
